# Single Administration of Ultra-Low-Dose Lipopolysaccharide in Rat Early Pregnancy Induces TLR4 Activation in the Placenta Contributing to Preeclampsia

**DOI:** 10.1371/journal.pone.0124001

**Published:** 2015-04-08

**Authors:** Pingping Xue, Mingming Zheng, Ping Gong, Caimei Lin, Jianjun Zhou, Yujing Li, Li Shen, Zhenyu Diao, Guijun Yan, Haixiang Sun, Yali Hu

**Affiliations:** 1 Drum Tower Clinical Medical College, Nanjing Medical University, Nanjing, China; 2 Department of Obstetrics and Gynecology, Nanjing Drum Tower Hospital, Nanjing University Medical School, Nanjing, China; 3 Nanjing Drum Tower Hospital Clinical College of Traditional Chinese and Western Medicine, Nanjing University of Chinese Medicine, Nanjing, China; 4 Southeast University Medical School, Nanjing, China; State Key Laboratory of Reproductive Biology, Institute of Zoology, Chinese Academy of Sciences, CHINA

## Abstract

Balanced immune responses are essential for the maintenance of successful pregnancy. Aberrant responses of immune system during pregnancy increase the risk of preeclampsia. Toll-like receptor 4 (TLR4) plays a crucial role in the activation of immune system at the maternal-fetal interface. This study aimed to generate a rat model of preeclampsia by lipopolysaccharide (LPS, a TLR4 agonist) administration on gestational day (GD) 5 as rats are subjected to placentation immediately after implantation between GDs 4 and 5, and to assess the contribution of TLR4 signaling to the development of preeclampsia. Single administration of 0.5 μg/kg LPS significantly increased blood pressure of pregnant rats since GD 6 (systolic blood pressure, 124.89 ± 1.79 mmHg *versus* 119.02 ± 1.80 mmHg, *P* < 0.05) and urinary protein level since GD 9 (2.02 ± 0.29 mg *versus* 1.11 ± 0.18 mg, *P* < 0.01), but barely affected blood pressure or proteinuria of virgin rats compared with those of saline-treated pregnant rats. This was accompanied with adverse pregnancy outcomes including fetal growth restriction. The expression of TLR4 and NF-κB p65 were both increased in the placenta but not the kidney from LPS-treated pregnant rats, with deficient trophoblast invasion and spiral artery remodeling. Furthermore, the levels of inflammatory cytokines were elevated systemically and locally in the placenta from pregnant rats treated with LPS. TLR4 signaling in the placenta was activated, to which that in the placenta of humans with preeclampsia changed similarly. In conclusion, LPS administration to pregnant rats in early pregnancy could elicit TLR4-mediated immune response at the maternal-fetal interface contributing to poor early placentation that may culminate in the preeclampsia-like syndrome.

## Introduction

Preeclampsia, as a pregnancy-specific disorder complicating 2–8% of pregnancy, is a leading cause of maternal and perinatal morbidity and mortality worldwide [1]. Although the ultimate cause of preeclampsia remains elusive, it has mainly been ascribed to poor placentation in early pregnancy, to which impaired spiral artery (SA) remodeling contributes as a major defect [1–3]. Balanced immune responses are essential for the maintenance of successful pregnancy. Aberrant responses of immune system during pregnancy are suggested to play an important role in the pathogenesis of pregnancy disorders with impaired placentation/remodeling, such as recurrent miscarriage, preeclampsia, and fetal growth restriction (FGR) [4–6].

Accumulating evidence shows that pathological stimuli may predispose immune maladaptation and abnormal placentation observed in preeclampsia, in which the toll-like receptor 4 (TLR4) is playing a vital role [7–9]. TLR4, a receptor known to be most abundantly expressed by trophoblast in the placenta, detects lipopolysaccharide (LPS) from gram-negative bacteria, and then activates nuclear factor-kappa B (NF-κB) to secrete cytokines, recruits unique immune cells, and facilitates the development of immunity at the maternal-fetal interface [10–12]. The expression of TLR4 was demonstrated increased in the placenta of women with preeclampsia [7]. Cytokines, such as tumor necrosis factor α (TNF-α), interleukin (IL)-6 and monocyte chemoattractant protein-1 (MCP-1), were increased systemically and locally in the placenta of preeclampsia-bearing women [13,14]. Our *in vitro* study found that LPS inhibited the invasion of human trophoblast cells [15]. Moreover, numerous animal studies revealed that administration of LPS to pregnant rats induced preeclampsia-like symptoms [16–19]. However, interventions in these animal studies were generally applied around gestational day (GD) 14 that have missed the early stage of placental development, which appears to be a pivotal time point in the development of preeclampsia [1–3].

The aim of this study was to generate a rat model of preeclampsia by an administration of ultra-low-dose LPS around early placentation, and to assess the contribution of TLR4 signaling to the development of preeclampsia. Concerning that placentation in the rat starts immediately after implantation between GDs 4 and 5 [20,21], pregnant rats were administrated with LPS on GD 5.

## Materials and Methods

### Animals and experimental protocol

This study was performed according to the guidelines of Experimental Animals Management Committee (Jiangsu Province, China) and ethics approval was obtained from the Animal Care and Use Committee of Nanjing Drum Tower Hospital (SYXK 2009–0017). Female Sprague-Dawley rats (10 weeks old) were raised in a light- and humidity-controlled room with free access to food and water. The experiment involved three groups: LPS-treated pregnant group (N = 15), saline-treated pregnant group (N = 15) and LPS-treated non-pregnant group (N = 5). Female rats were rendered pregnant by being housed on proestrus with fertile males at a 2:1 ratio overnight. The day on which pregnancy was confirmed by the presence of vaginal spermatozoa was designated as GD 0. For LPS-treated and saline-treated pregnant groups, the treatment (0.5 μg/kg LPS (*Escherichia coli* serotype 0111:B4, Sigma-Aldrich) dissolved in 2 mL saline, or corresponding volume of saline) was administrated through tail vein by an infusion pump (infusion rate, 2 mL/h) on GD 5. Additionally, corresponding LPS injection was performed on virgin rats (LPS-treated non-pregnant group). Blood pressure and urinary protein level were measured throughout pregnancy. The rats were euthanized by cervical dislocation under anesthesia on GD 18. Afterwards, the numbers of viable and resorbed pups were counted and recorded, and the wet weights of pups and placentas were measured and recorded.

### Patients, placenta and serum collection

This study was approved by the Scientific Research Ethics Committee of Nanjing Drum Tower Hospital (2009041) and written informed consent was obtained from each participant. Eligible cases were singleton pregnancies diagnosed as severe preeclampsia according to the American Congress of Obstetricians and Gynecologists criteria. Human placenta and serum were collected from normal pregnancy and gestationally matched pregnancy complicated by severe preeclampsia, snap-frozen and stored at -80°C until use.

### Cell culture

Immortalized trophoblast cell line HTR-8/SVneo was established and maintained as described previously [22,23]. After culture in RPMI 1640 (HyClone) with 2.5% FBS (HyClone) for 12 h, the cells were treated with a series of concentrations (0–200 ng/mL) of LPS in 10% FBS for 0–48 h. To investigate the relationship between NF-κB activation and cytokine secretion, the cells were cultured in the presence of LPS after treatment with pyrrolidine dithiocarbamate (PDTC, an inhibitor of NF-κB; Sigma-Aldrich) for 1 h. The cells were harvested for detection of NF-κB phosphorylated p65 (P-p65) and p65 protein, and supernatants were collected, centrifuged to remove cellular debris, and stored at -80°C for later analysis of cytokine secretion.

### Blood pressure and urinary protein level

The systolic blood pressure (SBP) of each rat (08:00 am-10:00 am) was monitored by tail-cuff plethysmography (BP-98A; Softron, Tokyo, Japan), which correlates well with telemetry measurement of SBP [24]. Briefly, each rat was warmed to 38°C and SBP was assessed continuously 15 times, in which 3 continuous values of variation of less than 6 mmHg were averaged to define maternal SBP.

The urine of each rat (20:00 pm-10:00 am) was collected every 3 days with dams housed individually in metabolic cages in the absence of food to eliminate contamination of urinary protein measurement by fallen food particles. Urinary protein level was measured using the pyrogallol red method [25].

### Assays of hemostatic parameters

Platelets of each rat were counted manually by two experienced technicians after lysis of erythrocyte with ammonium oxalate. Plasma level of AT-III was detected by immunoturbidimetry assay kit (Dade Behring) and that of D-dimmer was determined by immunoprecipitation sandwich kit on NycoCard Reader II analyzer (Axis-Shield PoC AS).

### Histology and immunostaining

Implantation site (uterus + placenta) from pregnant rats was embedded with paraffin and sections were cut step-serially from each implantation site parallel to the mesometrial-fetal axis. For each implantation site, one set of sections containing a central maternal arterial channel were selected for staining periodic acid-Schiff (PAS) as a fibrinoid tissue marker, cytokeratin as a trophoblast marker, and α-SMA as a vascular smooth muscle cell (VSMC) marker [26–28]. Trophoblast invasion and SA remodeling were evaluated on slides using HMIAS-2000 High Definition Color Image Analysis System (Qianping Image Technology Co., Ltd., Wuhan, China). The fibrinoid layer around SAs and/or the basal surface of cytokeratin-positive or α-SMA-positive cells were used as boundary with which the luminal contour of SA cross section in the mesometrial triangle was manually delineated, and the percentages of fibrinoid layer, cytokeratin-positive and α-SMA-positive cells of corresponding SA contour were calculated, respectively. TLR4 and NF-κB p65 expression in the placenta from pregnant rats and patients, and kidney from pregnant rats were determined using corresponding antibodies (TLR4, 1:100 dilutions, Abcam; NF-κB p65, 1:200 dilutions, Bioworld). Corresponding concentrations of anti-IgG served as non-specific controls. Additionally, kidney slices from rats were stained with hematoxylin and eosin by standard technique as previously mentioned [25].

### RNA isolation and quantitative real-time PCR

Total RNA was extracted from tissues using TRIzol reagent (Invitrogen) and 1 μg of purified RNA was reverse-transcribed to cDNA by a PrimeScript RT Master Mix kit (Takara). Reactions of quantitative real-time PCR (qRT-PCR) were performed as previously mentioned [29] and the specific primers for qRT-PCR are shown in S1 Table. Data were analyzed by the 2^–ΔΔCT^ method [30], and GAPDH was used for normalization.

### Western blot analysis

Proteins in the cells were prepared and separated by SDS-PAGE as previously described [23]. Immunoblotting was performed with primary antibodies raised against NF-κB P-p65 (1:1,000 dilutions; Cell Signaling), NF-κB p65 (1:1,000 dilutions; Santa Cruz) and GAPDH (1:5,000 dilutions; Bioworld). Immunodetection was accomplished using a goat anti-rabbit (1:5,000 dilutions; Bio-Rad Laboratories) secondary antibody, and an enhanced chemiluminescence detection kit (Millipore).

### Cytokine detection by enzyme-linked immunosorbent assay

Cytokines IL-6 and MCP-1 in rat serum, human serum and cell culture medium were quantitatively detected by enzyme-linked immunosorbent assay (ELISA) kits (human IL-6 (detection range, 0.37–1,000 pg/mL), human MCP-1 (detection range, 2.18–4,000 pg/mL) and rat IL-6 (detection range, 2.57–8,000 pg/mL): Sunny ELISA Kits, MultiSciences, Hangzhou, China; rat MCP-1 (detection range, 4.7–500 pg/mL): eBioscience) according to the manufacturers’ instructions.

### Statistical analysis

Data were presented as mean ± SEM from at least three independent experiments performed in triplicate. Differences between two means were determined using Student’s *t*-test. One-way ANOVA was performed for the comparison of multiple groups. In addition, data on litter size, FGR, fetal resorption and placenta abruption were analyzed using Wilcoxon signed-rank test. A *P*-value of less than 0.05 was considered significant.

## Results

### LPS administration to rats in early pregnancy induced a preeclampsia-like syndrome

By administrating pregnant rats with 0.5 μg/kg LPS on GD 5, a novel rat model of preeclampsia was established, which exhibited significant and characteristic hypertension, proteinuria and adverse pregnancy outcomes compared to the control pregnant group did.

#### Blood pressure

**Fig 1:**
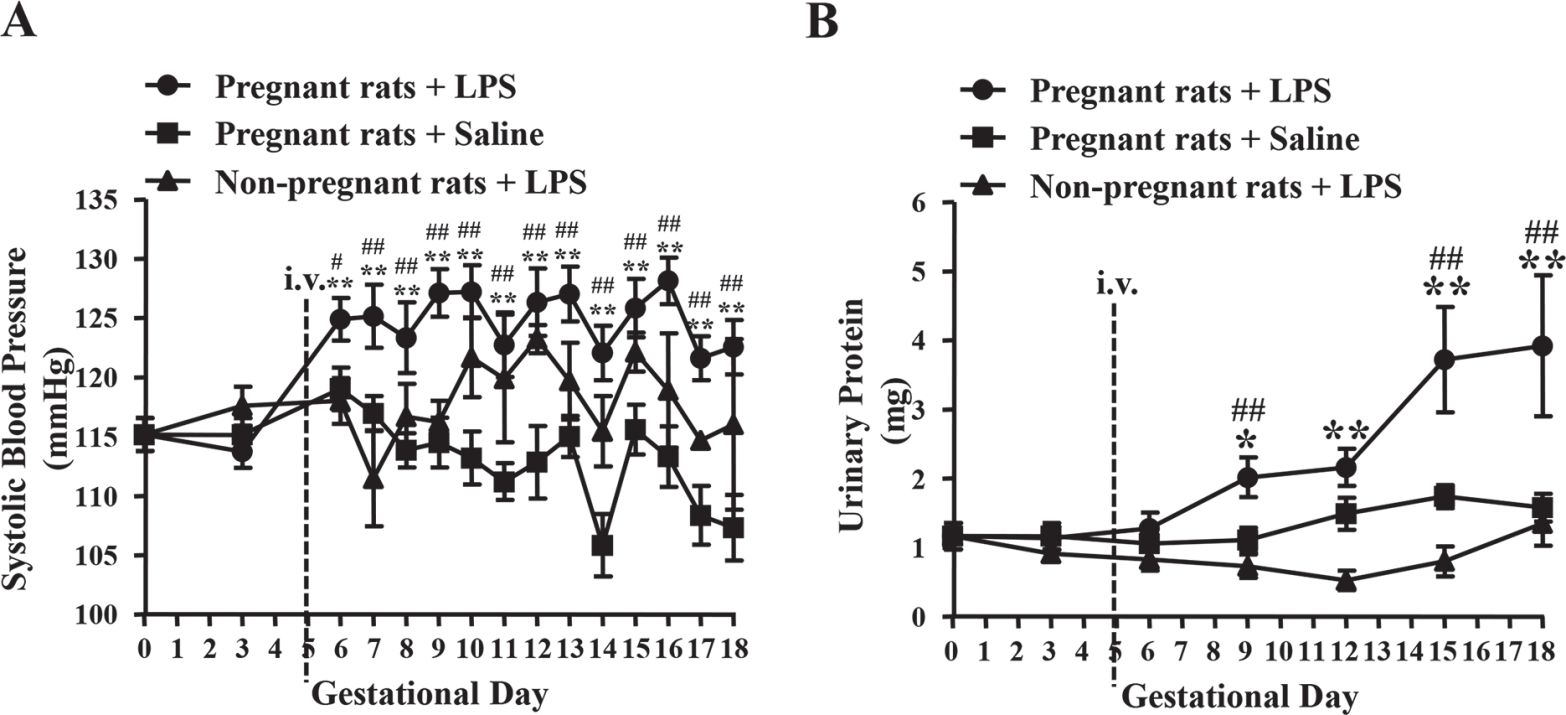
Mean SBP (A) and urinary protein level (B) of each group on different GDs. Pregnant rats + LPS, N = 15; Pregnant rats + Saline, N = 15; Non-pregnant rats + LPS, N = 5. Data are expressed as mean ± SEM, *P < 0.05, **P < 0.01 vs. GD 0; ^#^ *P* < 0.05, ^##^ *P* < 0.01 vs. saline-treated pregnant group on the corresponding GD.

There were no significant differences in SBP among the three groups before LPS infusion (Fig 1A). After LPS administration to pregnant rats on GD 5, SBP was significantly increased from GD 6 to 18 compared with that of the saline-treated pregnant group on the corresponding GD (GD 6, 124.89 ± 1.79 mmHg *versus* 119.02 ± 1.80 mmHg, *P* < 0.05) and prior to LPS infusion (from GD 3, 113.73 ± 1.39 mmHg to GD 18, 122.53 ± 2.31 mmHg, *P* < 0.01). However, SBPs of the saline-treated pregnant group and LPS-treated non-pregnant group did not change significantly throughout pregnancy.

#### Proteinuria

Urinary protein levels of the three groups were similar before LPS infusion (Fig 1B). After LPS administration to pregnant rats on GD 5, such level was significantly elevated from GD 9 to 18, relative to that of the saline-treated pregnant group on the corresponding GD (GD 9, 2.02 ± 0.29 mg *versus* 1.11 ± 0.18 mg, *P* < 0.01) and prior to LPS infusion (from GD 3, 1.17 ± 0.19 mg to GD 18, 3.92 ± 1.02 mg, *P* < 0.01). However, urinary protein levels of the saline-treated pregnant group or the LPS-treated non-pregnant group only changed moderately throughout pregnancy.

#### Pregnancy outcomes

The percentages of FGR, fetal resorption and placenta abruption in the LPS-treated pregnant group were significantly higher than those of the control pregnant group (*P* < 0.05). However, the two groups had similar number of live pups and placenta weight (Table 1).

**Table 1:** Pregnancy outcomes in different pregnant groups.

	Pregnant rats + Saline (N = 15)	Pregnant rats +LPS (N = 15)
Litter Size	13.40 ± 0.57	12.80 ± 0.73
FGR (%)	6 (12/200)	13.92 (27/194)**
Resorbed Fetus (%)	0 (0/200)	3.61 (7/194)*
Placenta Weight (g)	0.48 ± 0.01	0.48 ± 0.01
Placenta Abruption	0 (0/200)	4.12 (8/194)**

*P<0.05

**P<0.01, compared with the saline-treated pregnant group.

#### Hemostatic parameters

The platelet counts and plasma levels of AT-III and D-Dimmer of the three groups did not differ significantly (Table 2).

**Table 2:** Hemostatic parameters of each group on GD 18 (Mean ± SEM).

	Pregnant rats + Saline (N = 15)	Pregnant rats + LPS (N = 15)	Non-pregnant rats + LPS (N = 5)
Platelet (×10^9^/L)	850.13 ± 56.87	836.21 ± 75.42	525.33 ± 187.93
AT-III (%)	142.75 ± 1.97	141.61 ± 3.68	143.55 ± 14.45
D-Dimmer (mg/L)	0.06 ± 0.02	0.10 ± 0.06	0.04 ± 0.03

### LPS administration to rats in early pregnancy altered the expression of TLR4 and NF-κB p65 in the placenta

**Fig 2:**
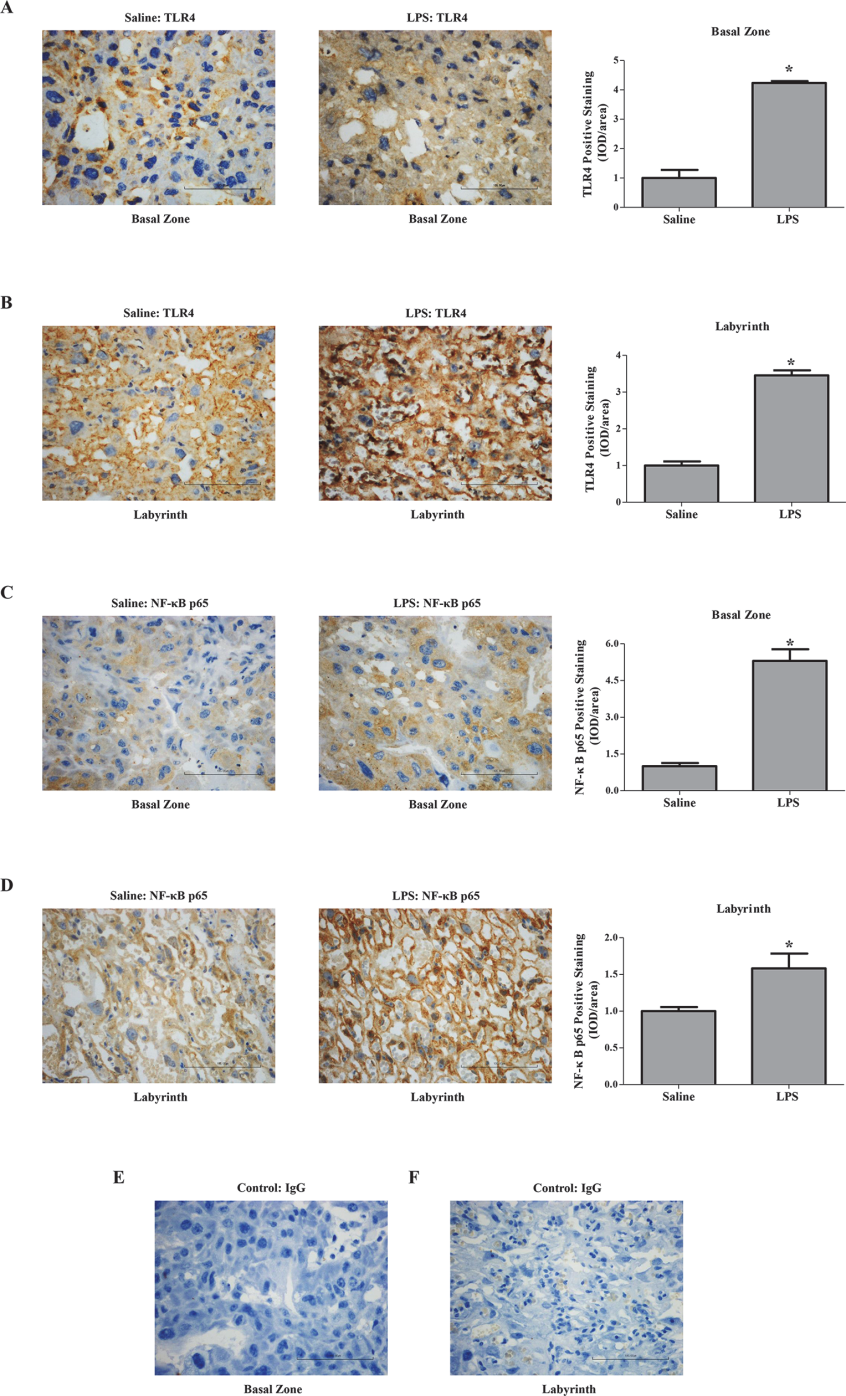
Expression of TLR4 and NF-κB p65 in the placenta of different pregnant groups. Immunohistochemistry for TLR4 (A-B) and NF-κB p65 (C-D) in paraffin-embedded placental tissue sections (4 μm) from the saline-treated and LPS-treated pregnant rats. Corresponding concentrations of anti-IgG served as non-specific controls (E-F). Data are expressed as mean ± SEM, N = 1–2 placentas for 10 rats/group, *P < 0.05 vs. saline-treated pregnant group. Magnification 400 ×. Bar 100 μm.

TLR4 in rat placenta was predominantly expressed by spongiotrophoblast cell, trophoblastic giant cell and glycogen cell in the basal zone (Fig 2A) as well as trophoblastic epithelium in the labyrinth (Fig 2B). In the pregnant group treated with LPS, TLR4 expression was significantly increased approximately 4.23- and 3.45-fold in the basal zone and labyrinth on GD 18 compared to that in the saline-treated pregnant group, respectively (*P* < 0.05) (Fig 2A and 2B). Meanwhile, significantly more NF-κB p65 was expressed in TLR4 positive cells in the placenta after LPS infusion (*P* < 0.05) (Fig 2C and 2D).

### LPS administration to rats in early pregnancy induced deficient trophoblast invasion and SA remodeling

**Fig 3:**
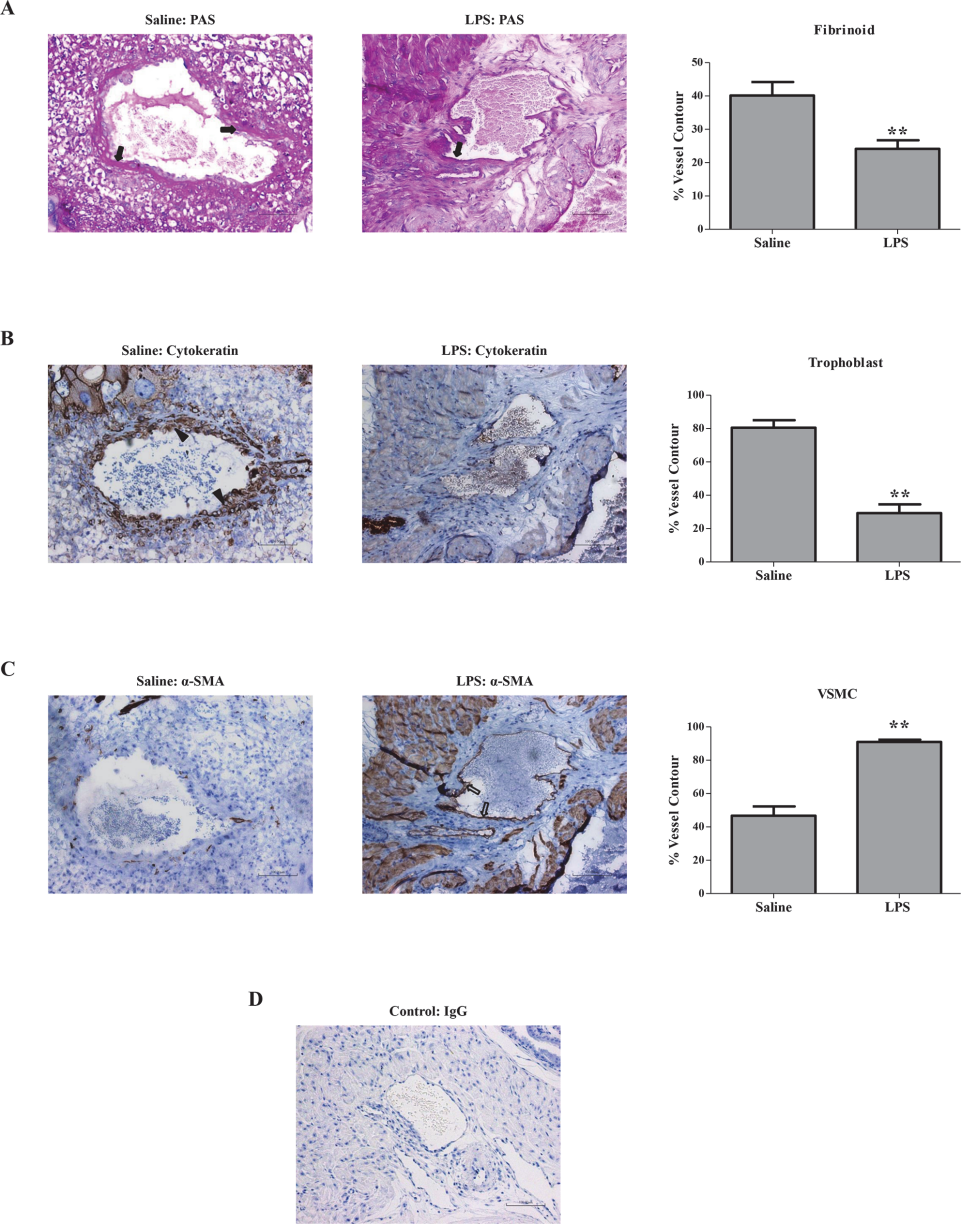
Trophoblast invasion and SA remodeling in different pregnant groups. Representative PAS staining of fibrinoid (solid arrows), cytokeratin staining of trophoblast invasion (solid arrowheads), and α-SMA staining of VSMC (hollow arrows) in the SA of mesometrial triangle. The percentages of fibrinoid (A), trophoblast (B) and VSMC (C) of total SA contour length were determined. Corresponding concentrations of anti-IgG served as non-specific controls (D).Data are mean ± SEM, 2–3 SAs measured from 1–2 implantation sites per rat, N = 10 rats per group. **P<0.01 vs. saline-treated pregnant group. Magnification 200 ×. Bar 100 μm.

Trophoblast invasion and trophoblast-mediated SA remodeling in the mesometrial triangle were assessed (Fig 3). PAS staining revealed decreased deposition of fibrinoid in the SA of the LPS-treated pregnant group compared with that in the control pregnant group (Fig 3A). Specifically, cytokeratin staining revealed that the LPS-treated pregnant group was significantly less prone to trophoblast invasion of SA than the control pregnant group (Fig 3B). However, significantly more of the SA contour was occupied by α-SMA-positive VSMCs in the LPS-treated pregnant group (Fig 3C). Hence, LPS administration to rats in early pregnancy undermined trophoblast invasion and SA remodeling.

### LPS administration to rats in early pregnancy induced renal alterations, not associated with alterations in the expression of TLR4 or NF-κB p65 in the kidney

**Fig 4:**
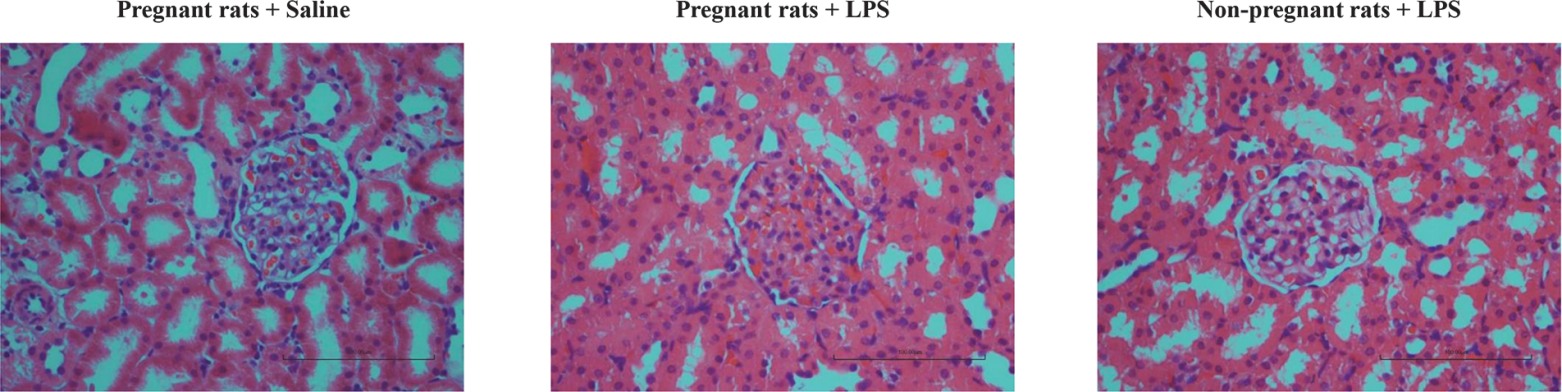
Morphological features of kidney in different groups. Kidney specimens from different groups were examined by hematoxylin and eosin staining. Magnification 400 ×. Bar 100 μm.

**Fig 5:**
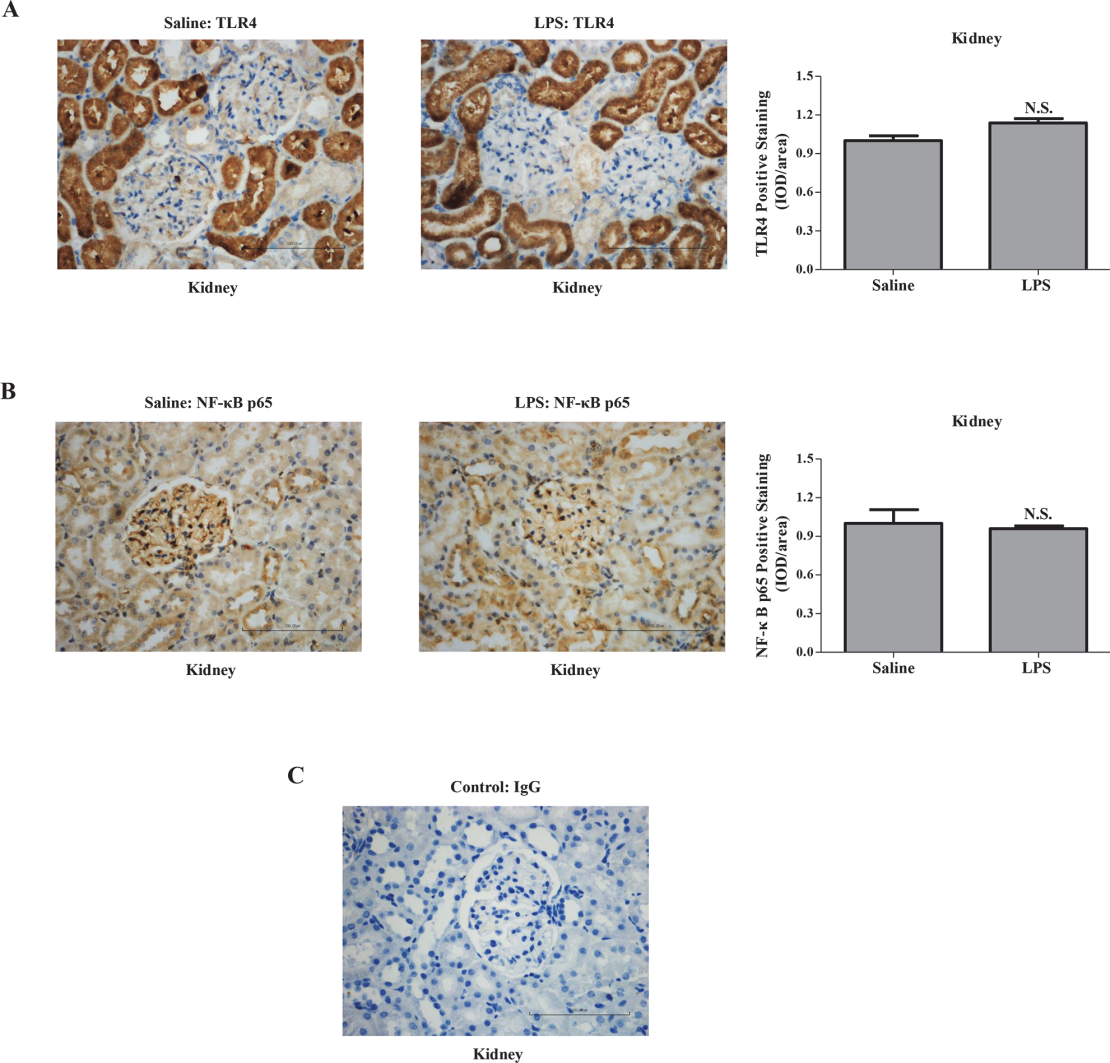
Expression of TLR4 and NF-κB p65 in the kidney of different pregnant groups. Immunohistochemistry for TLR4 (A) and NF-κB p65 (B) in paraffin-embedded renal tissue sections (4 μm) from the saline-treated and LPS-treated pregnant rats. Corresponding concentrations of anti-IgG served as non-specific controls (C). Data are expressed as mean ± SEM, N = 1 kidney for 10 rats/group, N.S. means *P* > 0.05. Magnification 400 ×. Bar 100 μm.

Histological examination of the kidney showed that LPS administration to rats in early pregnancy caused glomerular endothelial swelling with occlusion of capillary loops and urinary space (Fig 4). To determine whether renal morphological alterations were associated with the immune response of the kidney *per se*, the expression and location of TLR4 and its downstream target NF-κB p65 in the kidney were assessed by immunostaining (Fig 5). Predominantly expressed by tubular epithelial cell in the kidney, TLR4 had similar expression in the pregnant groups (Fig 5A). Similarly, the expression of NF-κB p65 was not significantly different in the kidney of the pregnant groups (Fig 5B).

### LPS administration to rats in early pregnancy promoted cytokine secretion in the placenta but not the kidney

**Fig 6:**
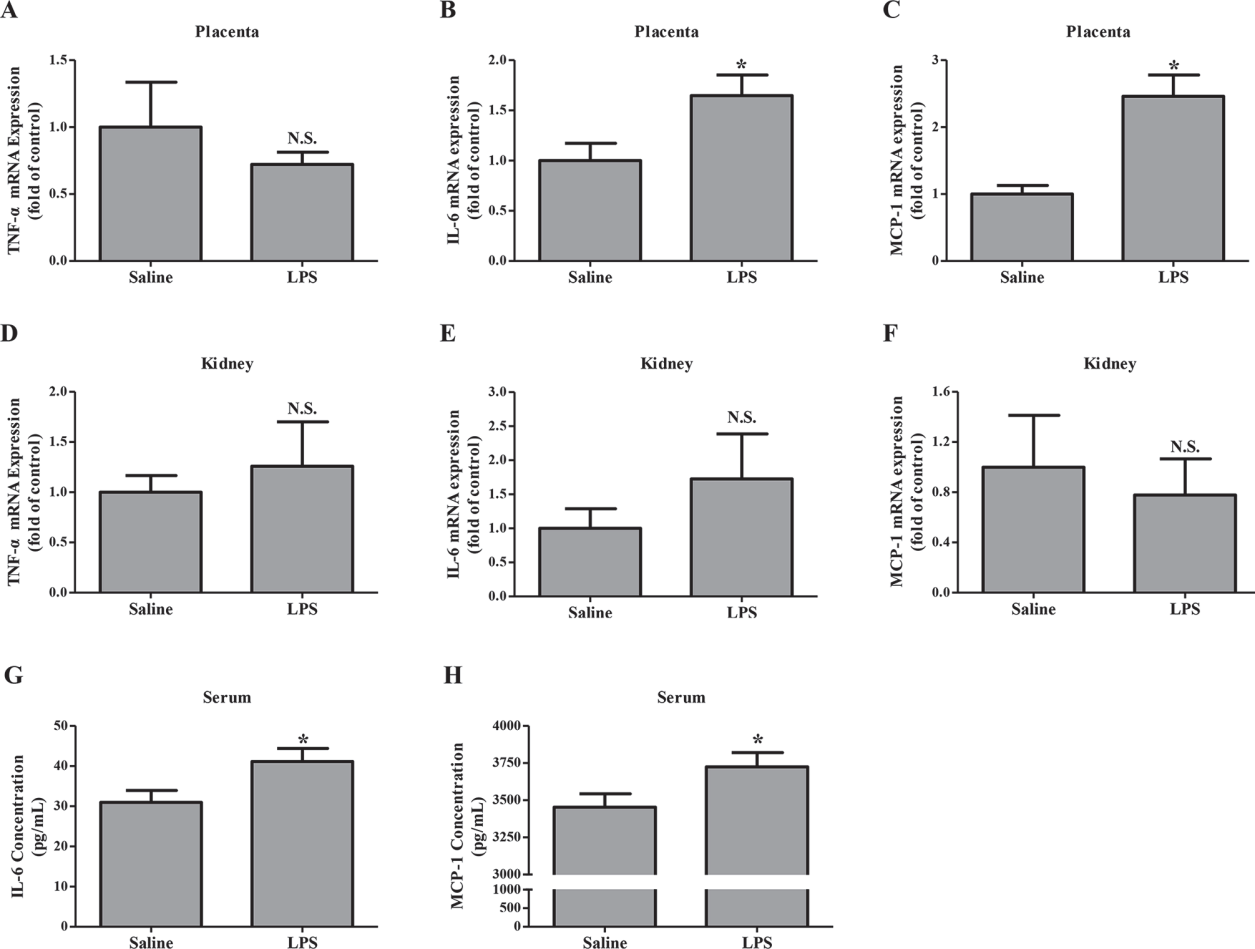
Cytokine production in different pregnant groups. (A-F), mRNA levels of TNF-α, IL-6 and MCP-1 in the placenta (A-C) and kidney (D-F) from different pregnant groups were measured by qRT-PCR and normalized to that of GAPDH. Data are mean ± SEM, N = 1–2 placentas for 10 rats/group and 1 kidney for 10 rats/group, *P < 0.05 vs. saline-treated pregnant group, N.S. means *P* > 0.05. (G-H), Concentrations of IL-6 (G) and MCP-1 (H) in the serum from different pregnant groups were measured by ELISA. Data are expressed by mean ± SEM, N = 10 rats per group. *P < 0.05 vs. saline-treated pregnant group.

Cytokine production in the placenta and kidney from pregnant groups were investigated by qRT-PCR on GD 18. The level of TNF-α mRNA in the placenta and kidney did not show significant differences between pregnant groups (Fig 6A and 6D). However, much stronger signals for both IL-6 and MCP-1 were observed in the placenta (*P* < 0.05) but not the kidney from the LPS-treated pregnant group than those from the pregnant control group (Fig 6B, 6C, 6E and 6F). ELISA further showed that serum IL-6 and MCP-1 in the LPS-treated pregnant group significantly exceeded those in the pregnant control group (*P* < 0.05) (Fig 6G and 6H).

### Circulating cytokines increased in preeclampsia-bearing women with high placenta TLR4/NF-κB p65 levels

**Fig 7:**
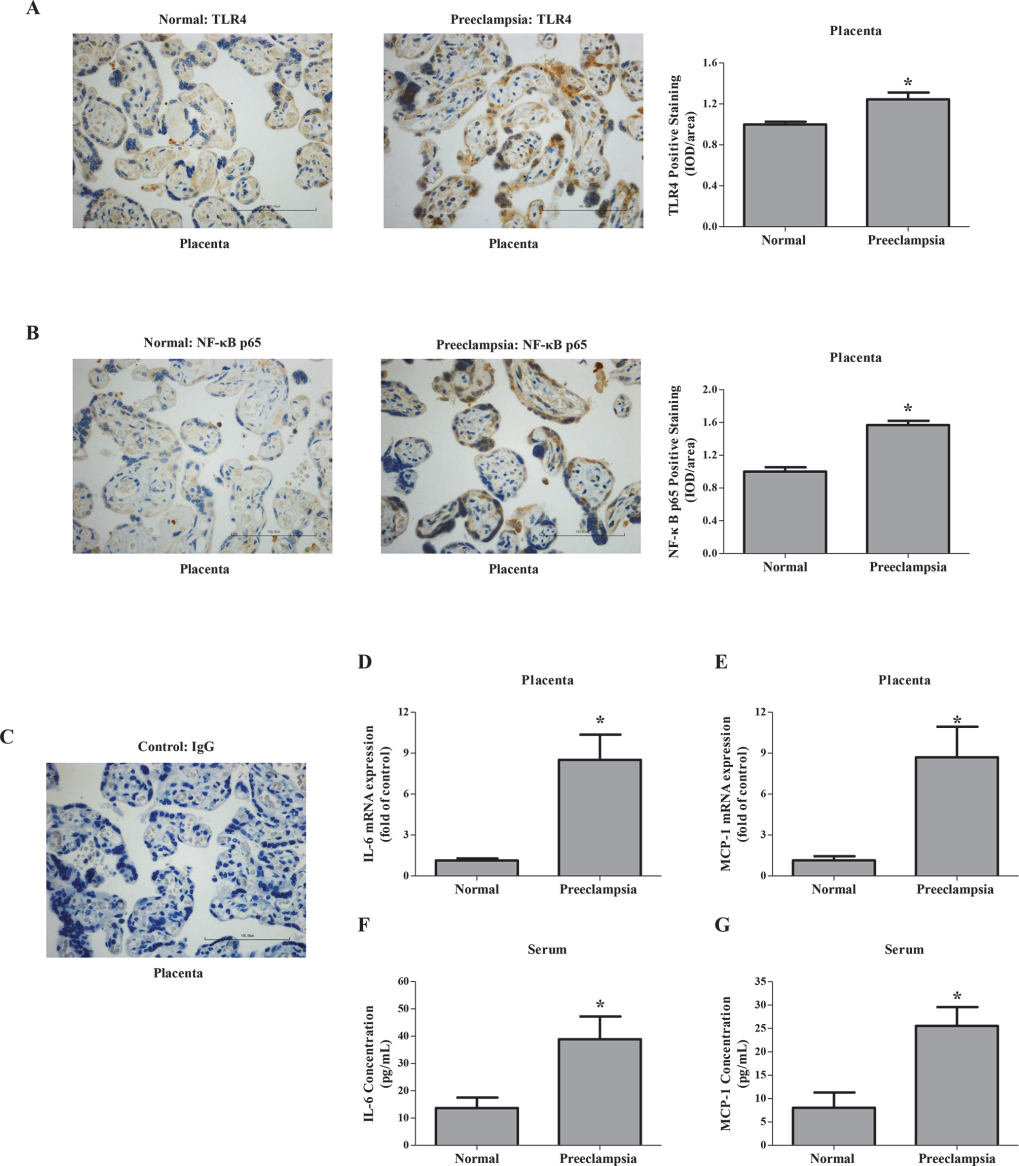
Expression of TLR4, NF-κB p65 and cytokines in human normal pregnancy and preeclampsia. (A-C), Immunohistochemistry for TLR4 (A) and NF-κB p65 (B) in paraffin-embedded placental tissue sections (4 μm) from human normal pregnancy and preeclampsia. Corresponding concentrations of anti-IgG served as non-specific controls (C). Magnification 400 ×. Bar 100 μm. (D-E), mRNA levels of IL-6 (D) and MCP-1 (E) in the placenta from normal pregnancy and preeclampsia. IL-6 and MCP-1 mRNA levels were measured by qRT-PCR and normalized to that of GAPDH. (F-G) Serum levels of IL-6 (F) and MCP-1(G) in human normal pregnancy and preeclampsia were quantitatively measured by ELISA. Data are expressed by mean ± SEM, N = 10. *Means *P* < 0.05.

Differences in the expression of TLR4 and its downstream target NF-κB p65 between the control and severe preeclampsia placenta were examined by immunostaining (Fig 7A and 7B). As shown in Fig 7A, TLR4 was predominantly expressed by syncytiotrophoblast in the term placental villi. TLR4 expression in severe preeclampsia placenta significantly increased in comparison to that in the control. Meanwhile, TLR4 positive trophoblasts showed higher levels of NF-κB p65 (Fig 7B).

In addition, both IL-6 and MCP-1 mRNA levels of severe preeclampsia placenta were significantly higher than those of the control (Fig 7D and 7E). ELISA further showed that serum concentrations of IL-6 and MCP-1 in severe preeclampsia significantly exceeded those of normal pregnancy (Fig 7F and 7G).

### LPS promoted cytokine secretion in human trophoblast cells through activation of NF-κB

**Fig 8:**
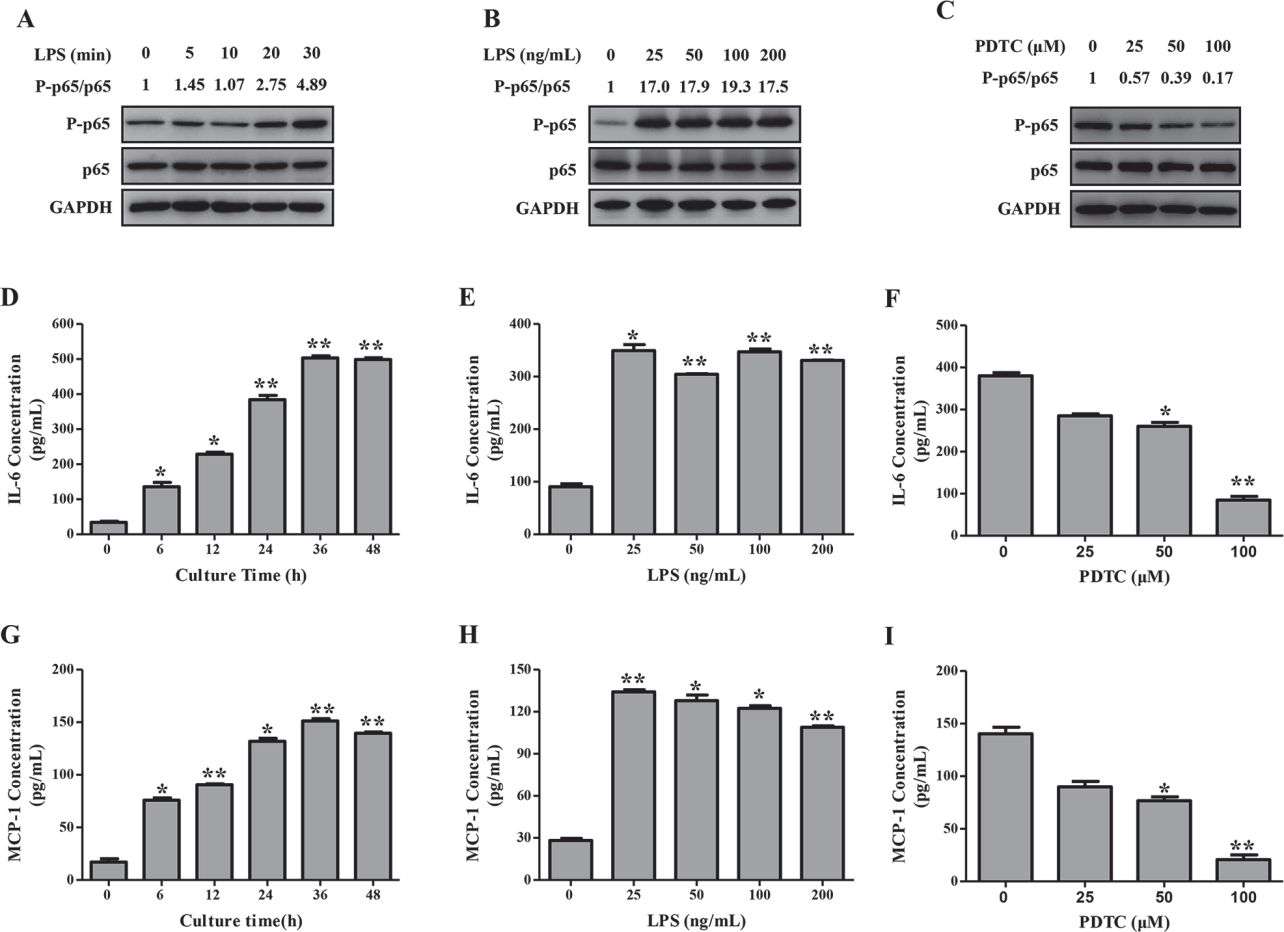
NF-κB activation and cytokine secretion following LPS stimulation in HTR-8/SVneo cells. NF-κB P-p65 and p65 levels were detected by Western blotting (A-C), and IL-6 and MCP-1 secretion were determined by ELISA (D-I) following LPS (100 ng/mL) stimulation for indicated times (A, D, G), or indicated concentrations of LPS stimulation for 30 min (B) or 24 h (E, H), or LPS (100 ng/mL) stimulation for 30 min (C) or 24 h (F, I) after pre-treatment with PDTC (0–100 μM). Results are shown as mean ± SEM of three separate experiments (*P < 0.05; **P < 0.01).

NF-κB activation *in vitro*, determined as the ratio of P-p65/p65, responded to LPS stimulation in a time-dependent manner. NF-κB activation was significantly increased 20 min after LPS stimulation (100 ng/mL) and remained thereafter till 30 min (Fig 8A). Similarly, NF-κB activation 30 min after treatment was enhanced depending on the dose of LPS but peaked at 100 ng/mL (Fig 8B). Meanwhile, LPS boosted the secretion of IL-6 and MCP-1 time-dependently and dose-dependently (Fig 8D, 8E, 8G and 8H). Moreover, pretreatment of trophoblasts with PDTC dose-dependently reduced LPS-induced secretion of IL-6 and MCP-1 (Fig 8F and 8I). Decreased production of cytokines was accompanied by attenuated activation of NF-κB (Fig 8C).

## Discussion

In this study, we successfully generated a novel animal model of preeclampsia by administrating 0.5 μg/kg LPS to pregnant rats on GD 5. The LPS treated-rats exhibited all the symptoms and pathological changes in the placenta that happened in human preeclampsia. We found that LPS triggered an immediate high blood pressure and remained the status until to the end of gestation in pregnant, but not in non-pregnant rats. The process is the same as the phenomenon observed on preeclampsia-like syndrome in the pregnant rats that exposed to LPS at late gestational ages [16]. LPS also significantly increased proteinuria in pregnant rats, which kept rising toward the end of pregnancy, mimicking another key symptom of human preeclampsia [3]. Giving the fact that placenta forms immediately after implantation between GDs 4 and 5 in rat [20], LPS was administrated to pregnant rats on GD 5, human preeclamptic phenotype on maternal-fetal interface, deficient trophoblast invasion and SA remodeling, were first generated in this rat model of preeclampsia. Like human being, preeclampsia is clinically manifested at or after 20 week gestation [1], while the abnormal placentation in this disease could start from the first trimester [6]. Since all these preeclampsia-like symptoms were only induced in pregnant rats, we believe that placenta is necessary for the onset of preeclampsia in both animal model and human. Meanwhile, inflammation factors might specifically trigger the release of placental factors.

Systemic exposure to LPS elicits a series of signal transductions that culminate in the release of numerous inflammatory mediators including cytokines [31]. Several of these cytokines have been involved in the delicate immune system balance at the maternal-fetal interface [12]. Among of them, TLR4 is a major contributor to the development of human preeclampsia. First, TLR4 was abundantly expressed in rat placenta (Fig 2); second, treated pregnant rats with its agonist, LPS, TLR4 expression was significantly up-regulated for approximately 4.23- and 3.45-fold in the basal zone and labyrinth on GD 18. Interestingly, increased TLR4 expression was found in the placenta from the patients with preeclampsia [7]. Furthermore, in our study, IL-6 level was elevated systemically and locally in the placenta, which might also contribute to the onset of maternal syndrome in the LPS-treated pregnant rats. However, the level of TNF-α produced by placenta remained in the LPS-treated pregnant group, as is the case in a rat model of preeclampsia performed by Fass et al. [17]. Additionally, elevated level of placental MCP-1 may contribute to the recruitment and activation of immune cells in the placenta [32].

As the downstream of LPS signaling, TLR4/NF-κB signal pathway might involve multiple symptoms of preeclampsia. On the blood pressure regulation, overexpression TLR4 down-regulated the levels of endothelial nitric oxide synthase (eNOS) and nitric oxide in mice endothelial cells [33]. This was consistent with our previous study showing decreased eNOS expression in human umbilical vein endothelial cells treated with LPS [34]. *In vivo* studies have also demonstrated that the expression of eNOS was decreased in the heart, lung, and aorta of rats injected with LPS [35]. TLR4 was also abundantly expressed in the rat kidney (Fig 5), whereas its expression in the kidney of pregnant groups did not differ significantly. The expression of TLR4 and its downstream target NF-κB p65 were both increased in the placenta from LPS-treated pregnant rats, which mimicked the changes observed in human placenta from patients with preeclampsia [7,15]. Anton et al. revealed that LPS stimulation induced cytokine expression in human trophoblast cells [36]. Consistently, LPS stimulation herein induced the secretion of inflammatory cytokines in the trophoblasts, which could be partially blocked by an NF-κB inhibitor, PDTC. Furthermore, we have previously found that LPS managed to inhibit trophoblast invasion [15]. In the present study, LPS administration to pregnant rats not only activated the placental TLR4/NF-κB pathway to secrete cytokines, but also resulted in inadequate trophoblast invasion and SA remodeling. Immune system at the maternal-fetal interface displays substantial modulatory effects on the remodeling of SA [5]; concomitantly, adequate SA remodeling plays a pivotal role in the delicate immune system balance at the maternal-fetal interface [37]. Upon LPS administration to pregnant rats, the immune system and SA remodeling are capable of potentiating each other leading to a vicious cycle.

In summary, we successfully established a new rat model of preeclampsia by single administration of ultra-low-dose LPS upon placentation, in which the activation of placental TLR4 signaling in response to LPS elicited deficient trophoblast invasion and SA remodeling contributing to poor placentation that may reach maximum intensities in the preeclampsia-like syndrome.

## Supporting Information

S1 Table Oligonucleotide primer sequences for qRT-PCR. (PDF)
