# Supplementary material for: Single Administration of Ultra-Low-Dose Lipopolysaccharide in Rat Early Pregnancy Induces TLR4 Activation in the Placenta Contributing to Preeclampsia
Source: PLoS One. 2015 Apr 8;10(4):e0124001. doi: 10.1371/journal.pone.0124001 (PMC4390151; doi:10.1371/journal.pone.0124001)
Supplement: S1 Table — (PDF) [file pone.0124001.s001.pdf]

Supporting Information

S1 Table. Oligonucleotide primer sequences for qRT-PCR.

Gene	Forward primer (5’→3’)	Reverse primer (5’→3’)
**Rat gene**		
TNF-α	CGTCGTAGCAAACCACCAAG	CACAGAGCAATGACTCCAAAG
IL-6	GCCCTTCAGGAACAGCTATG	CAGAATTGCCATTGCACAAC
MCP-1	TTCACAGTTGCTGCCTGTAG	TCTGATCTCACTTGGTTCTGG
GAPDH	ATGGGAAGCTGGTCATCAAC	GGATGCAGGGATGATGTTCT
**Human gene**		
IL-6	TACCCCCAGGAGAAGATTCC	TTTTCTGCCAGTGCCTCTTT
MCP-1	CCCCAGTCACCTGCTGTTAT	AGATCTCCTTGGCCACAATG
GAPDH	AACGGATTTGGTCGTATTG	GGAAGATGGTGATGGGATT
